# Development and Validation of the Occupational Therapy Engagement Scale for Patients with Stroke

**DOI:** 10.1155/2019/3164254

**Published:** 2019-03-06

**Authors:** Tzu-Yi Wu, Bella Ya-Hui Lien, Anthony H. Lequerica, Wen-Shian Lu, Ching-Lin Hsieh

**Affiliations:** ^1^Institute of Economics, Academia Sinica, Taipei, Taiwan; ^2^Department of Occupational Therapy, College of Medical and Health Science, Asia University, Taichung, Taiwan; ^3^Department of Business Administration, National Chung Cheng University, Jiayi Shi, Taiwan; ^4^Department of Physical Medicine and Rehabilitation, New Jersey Medical School-Rutgers, The State University of New Jersey, USA; ^5^Center for TBI Research, Kessler Foundation, New Jersey, USA; ^6^School of Occupational Therapy, Chung Shan Medical University, Taichung, Taiwan; ^7^School of Occupational Therapy, College of Medicine, National Taiwan University, Taipei, Taiwan; ^8^Department of Physical Medicine and Rehabilitation, National Taiwan University Hospital, Taipei, Taiwan

## Abstract

**Background/Aim:**

Almost all interventions in occupational therapy require the active engagement of the patients. However, no scale has been specifically designed for assessing engagement in occupational therapy. The purposes of this study were to develop the occupational therapy engagement scale (OTES) and to examine its unidimensionality, reliability, and predictive validity.

**Methods:**

The OTES was developed through the review of similar scales, eight experts' opinions, cognitive interviews, and pilot testing. The unidimensionality was verified with Rasch model fitting and principal component analysis. The Rasch reliability was also estimated. Pearson's correlation coefficient (*r*) was used to validate the predictive validity by examining the association between the Rasch scores of the OTES and patients' performance of activities of daily living (ADL).

**Results:**

A total of 253 patients with stroke were rated by 22 therapists using the OTES. The mean age of the patients was 62.3 ± 13.2 years old, and 65.2% of the patients were male. The infit and outfit MNSQ of the 12 items of the OTES ranged from 0.62 to 1.34. The unexplained variance of the first dimension of the principal component analysis was 4.0%. The mean person reliability of the OTES was 0.88. Pearson's *r* between the OTES and patients' ADL performance was 0.37.

**Conclusions:**

The results of Rasch analysis supported that the items of the OTES were unidimensional. The OTES had sufficient person reliability and predictive validity in patients with stoke.

## 1. Introduction

Almost all interventions in occupational therapy require the active engagement of the patients. A patient who is more engaged in occupational therapy may expend more effort, show better compliance, and want to engage in therapeutic activities more actively. Poor engagement in occupational therapy may lead to at least two consequences: a cessation of treatments and an insufficient amount of practice. Both consequences are likely to be detrimental to the patient's recovery from stroke, such as less functional gain and longer length of stay [1, 2]. Ultimately, low engagement in occupational therapy may decrease the efficacy of occupational therapy.

Once a patient has been assessed as having low engagement in occupational therapy, the therapist can review the results in order to identify the items that are the most problematic. For example, the therapist may reconfirm the patient's attention and comprehension, treatment goals, the difficulty of therapeutic activities, and previous occupation to identify the areas of weakness and tailor the intervention to meet the patient's needs. Then, the patient may understand that the therapeutic programs are designed to reach his/her goals and may be more willing to actively engage in the therapeutic activities. Thus, there is a need for therapists to evaluate the levels of patients' engagement in occupational therapy.

In this study, patients' engagement in occupational therapy is defined as patients' commitment to therapeutic activities during occupational therapy sessions. According to the model for therapeutic engagement in rehabilitation [3], patients' engagement in rehabilitation can be affected by four factors: the patients' willingness, capability, social environment, and physical environments. Moreover, inconsistencies in the treatment goals between therapists and patients may result in low engagement because the patients may think the therapeutic activities are meaningless. The above factors may change as time after the stroke onset; it would be helpful for occupational therapists to monitor patients' engagement in occupational therapy regularly. Then therapists can manage the engagement and outcomes accordingly. To monitor patients' engagement in occupational therapy accurately, a scale with sound validity and reliability to assess engagement is crucial.

To the best of our knowledge, engagement in therapy in a rehabilitation context can be assessed with 3 scales: the Pittsburgh Rehabilitation Participation Scale (PRPS) [4], the Hopkins Rehabilitation Engagement Rating Scale (HRERS) [5], and the Rehabilitation Therapy Engagement Scale (RTES) [6]. However, each of the three scales has drawbacks. The PRPS has only one item, and the ratings combine multiple aspects of engagement (e.g., attendance, effort, completion of activities, and need for encouragement), making it difficult to obtain comprehensive information on patients' engagement. The HRERS has only 5 items, a number too small to cover all the important domains of engagement in occupational therapy (e.g., cooperating with the therapist and following the therapist's instructions). The RTES has 15 items and good interrater reliability [6]. Nevertheless, some items on the RTES have similar/redundant concepts (e.g., “Focuses concentration intensely on therapy exercises during the session” and “Sustains attention to follow through on tasks until completed”), and some items contain more than one question in a single item. For example, the item “Puts forth effort, works diligently, and strives for accuracy on all tasks” (item 8 of the RTES) contains three questions: (1) puts forth effort on all tasks, (2) works diligently on all tasks, and (3) strives for accuracy on all tasks. These items need to be revised to avoid confusing raters. In addition, two items of the RTES do not belong to the same construct as other items when applied in an occupational therapy setting [6], so it would be inappropriate to sum the scores of these two items with the other items of the RTES.

There are two existing scales that evaluate patients' general performance in occupational therapy with items of patients' engagement in occupational therapy [7, 8]. The two scales are the occupational therapy task observation scale (OTTOS) and the comprehensive occupational therapy scale (COTE scale). Yet these two scales contain insufficient numbers of items to assess patients' engagement. The OTTOS has four items, and the COTE scale has five items. Using such small numbers of items to assess patients' engagement has at least two disadvantages. First, these two measures do not contain some important concepts regarding patients' engagement in occupational therapy, such as attitude toward executing therapeutic programs and quality of execution of therapeutic activities. Second, a small number of items would result in limited reliability [9, 10]. These two disadvantages diminish the application of the scores for making further clinical decisions.

In summary, the scales mentioned above either have limited items for assessing engagement or require revision of the descriptions of the items. It would not be proper to apply them to monitor patients' engagement in occupational therapy regularly. The purposes of this study were to develop the occupational therapy engagement scale (OTES) and to validate the unidimensionality (one type of construct validity), reliability, and predictive validity of the OTES in patients with stroke.

## 2. Method

### 2.1. Research Design

To correct the flaws of existing measures (i.e., not capturing the full scope of patients' engagement, containing redundant items, items containing more than one question, and items belonging to other dimensions), we tried to address the flaws in the following two phases. In Phase 1, the development of the OTES, the first three flaws were addressed during “item construction” and “expert committee review.” In Phase 2, we conducted a cohort study to examine the unidimensionality (to address the last flaw), Rasch reliability, and predictive validity of the OTES. The predictive criterion of the OTES was patients' performance of activities of daily living (ADL). The reason was that most patients with stroke visit an occupational therapist because they have difficulties returning to daily life after stroke [11, 12]. Through engaging in occupational therapy programs designed to promote ADL performance [13], the patients should become more independent in daily life. Therefore, we assumed that patients would have better ADL performance after actively engaging in occupational therapy programs. This study was approved by the Institutional Review Board of National Taiwan University Hospital and that of Chung Shan Medical University Hospital.

### 2.2. Phase 1: Development of the OTES

#### 2.2.1. Subjects

The participants included occupational therapists and their patients with stroke. Occupational therapists were recruited if they met the following criteria: (1) more than 6 months of experience in working in adult physical dysfunction settings and (2) experience in treating patients with stroke. Inpatients who were in subacute care (receiving rehabilitation) after stroke were recruited if they met the following criteria: (1) diagnosis of stroke, (2) history of at least 6 occupational therapy treatments with a therapist, and (3) ability to follow one-step verbal instructions. Patients with stroke were excluded if they had other major comorbidities (e.g., cancer and Alzheimer's disease).

#### 2.2.2. Procedure

The development of the OTES included three steps:

1. Item construction: we primarily adopted the items of the RTES, which contains more comprehensive concepts of patients' engagement than other rehabilitation engagement scales. The RTES was developed for patients with acquired brain injuries [6], who may have symptoms in common with patients with stroke. The revision was approved by the developer of the RTES. We revised the items according to four principles: (a) the items reflect the patients' engagement in occupational therapy, (b) the items fit the local cultural and occupational therapy settings, (c) all the items are distinct from each other, and (d) each item contains only one question. The items constructed in this step were named the OTES draft-1
2. Expert committee review: eight occupational therapists served on an expert committee to review the OTES draft-1 to ensure that the items (a) fit the contexts of occupational therapy and local culture, (b) included the entire scope of patients' engagement in occupational therapy programs, (c) described observable behaviors, and (d) were easy to understand. The experts were asked to add new items to fully cover the scope of patient engagement in occupational therapy according to their experience. All items were designed to reflect patients' engagement in occupational therapy. Two authors (the first and third authors) revised the items of the OTES draft-1 according to the committee's suggestions and discussed the revisions with the committee until the committee agreed with the revisions to produce the OTES draft-2
3. Cognitive interview: we recruited 14 occupational therapists who had not participated in the previous two steps to test the OTES draft-2 and recorded difficulties that occurred during the evaluation of patients' occupational therapy engagement (e.g., any confusion caused by the descriptions of the items, the format of the questionnaire, or the rating criteria). Before the therapists administered the OTES draft-2, we provided them with the manual of the OTES draft-2 to help them understand the scoring criteria and acquire sufficient knowledge about patient engagement. Two authors (the first author and the corresponding author) conducted cognitive interviews to determine the therapists' interpretations of the OTES draft-2 and to collect suggestions for revising the OTES draft-2 during field testing [14, 15]. After revising the OTES draft-2, we conducted further pilot testing to ensure that no further revisions were proposed

### 2.3. Phase 2: Validation of the OTES

#### 2.3.1. Subjects

We recruited a convenience sample of patients with stroke who received occupational therapy services at two medical centers from January 2, 2015, to January 31, 2016. The criteria for recruiting patients and therapists were the same as those in Phase 1.

#### 2.3.2. Procedure

The recruited patients were evaluated with the OTES by their occupational therapists after one week of daily intervention sessions. All therapists were provided with the manual of the OTES so that they would have sufficient knowledge for rating the scores. Regarding the timing of predictive criteria evaluations, the patients were evaluated by one of the four research assistants with the activities of daily living computerized adaptive testing system (ADL CAT) two months after discharge [16]. The patients' demographic data and medical history were collected from medical charts. All occupational therapists who participated in this study received 2 hours of training on how to administer the OTES.

#### 2.3.3. Measurement Tools

*(1) Occupational Therapy Engagement Scale (OTES)*. The OTES was developed as described previously.

*(2) Activities of Daily Living Computerized Adaptive Testing System (ADL CAT)*. The ADL CAT is a computerized adaptive test of performance of ADL (i.e., basic self-care activities, such as bathing or dressing) and instrumental ADL (i.e., advanced living skills, such as preparing meals) in patients with stroke [16]. The ADL CAT contains 34 items in the item bank and can be administered on a digital device (e.g., a smart phone) via the Internet. It has been shown that the ADL CAT has good reliability and good concurrent validity with the combined score of the Barthel Index and the Frenchay Activities Index [16].

## 3. Measurement Scheme for Classifying Aphasia

To characterize the participants' level of aphasia, the authors developed a measurement scheme to classify aphasia using the following criteria: (1) comprehension impairment: normal—no difficulty in understanding the conversation, mild—a few difficulties in comprehending the conversation (e.g., inability to understand long sentences or faster talking), moderate—comprehension of only short sentences or key words in the conversation, and severe—no comprehension of the conversation. In addition, the patients with severe comprehension impairment were still able to engage in therapeutic activities through therapists' demonstrations and repeated practice. (2) Expression impairment: normal—no difficulty in expressing themselves, mild—a few difficulties in expressing themselves (e.g., inability to talk fluently or to recall several words), moderate—ability to say only short sentences or keywords in the conversation, and severe—inability to talk. Although the patients with severe expression impairment were unable to speak, they could still understand conversations.

### 3.1. Data Analysis

#### 3.1.1. Descriptive Analysis

The score range and distribution of the OTES were examined. The floor and ceiling effects were also examined. The floor effect was the percentage of patients with the lowest possible score, whereas the ceiling effect was the opposite extreme [17]. Floor or ceiling effects exceeding 20% were significant [18].

### 3.2. Validation of the OTES

#### 3.2.1. Unidimensionality and Reliability

The partial credit model of Rasch analysis was applied to investigate the unidimensionality of the OTES because the descriptions of the response categories were different in several items [19, 20]. We assumed that patients' engagement in occupational therapy was unidimensional because patients' engagement in rehabilitation was validated as unidimensional in a previous study [6]. Through a set of generalized linear models and statistical procedures, we connected patients' level of engagement and the difficulty for a patient to achieve each item's criterion. Patients with higher engagement would have a higher probability of meeting the criteria of more difficult items. Infit and outfit mean squares (MNSQ) were used to ascertain data-model fitting. The item was removed if the infit or outfit MNSQ value was outside the appropriate range (0.6–1.4), which indicates that the item does not belong to the same dimension [21]. If any item was removed, we reconducted the Rasch analysis. In addition, we employed principal component analysis (PCA) of residuals to further determine the unidimensionality of the OTES. The variance of residuals of the PCA was used to determine whether other dominant dimensions existed in the OTES. The PCA of a residual was acceptable when no other dimensions explained the >10% variance of the residuals [22].

Person reliability coefficients were also calculated from the Rasch analysis. A coefficient ≥ 0.7 was considered adequate for using the sum score of the OTES for group comparisons (e.g., comparison of groups' mean scores of the OTES), whereas a coefficient ≥ 0.9 was adequate for individual comparisons (e.g., comparison of two individuals' sum scores of the OTES) [23].

The raw sum scores of the OTES could be transformed into Rasch scores (also known as logit scores) if its items fit the Rasch model's expectations. Every raw sum score would have a corresponding Rasch score no matter what the combination of the responses was. All Rasch analyses were performed in the Winsteps computer program (version 3.64.2).

#### 3.2.2. Appropriateness of Response Categories

We examined the appropriateness of the response categories of each item of the OTES by checking the order of the step difficulties (the threshold for two adjacent response categories) for each item. The response categories were considered appropriate when the step difficulties fit two criteria: (1) the step difficulties were in the same order as the intended response category order (i.e., no disordering); (2) the difference between adjacent step difficulties was 1.4–5.0 logits [24].

#### 3.2.3. Person-Item Mapping

We estimated the levels of patients' engagement in occupational therapy and the difficulty of the OTES items by Rasch analysis. We verified whether the items of the OTES matched the patients' levels of engagement in occupational therapy (person-item mapping) by using two examinations. First, we compared the range of levels of patients' engagement in occupational therapy levels and that of the item response difficulties. The range of item difficulties was sufficient when it covered the full range of patients' levels of engagement in occupational therapy. Second, we examined whether substantial gaps existed between the items' levels of difficulty. A gap was notable when it was equal to or larger than 0.5 logit (the unit of item difficulty) [25]. A gap indicates a lack of items estimating a patient's ability (e.g., level of engagement in OT in this study) within the gap. This lack of items may decrease the reliability of the estimation of a patient's ability.

#### 3.2.4. Predictive Validity

Predictive validity was examined using Pearson's *r* to examine the relationship between the Rasch scores of the OTES and scores of the ADL CAT at 2 months after discharge. To demonstrate acceptable predictive validity, the scores of the OTES should have at least low correlation (Pearson's *r* > 0.3) with those of the ADL CAT.

## 4. Results

### 4.1. Phase 1: The Development of the OTES

In step 1 (item construction), we rephrased all 15 items of the RTES and added the words “therapeutic activities” and/or “therapist” to some items. Although 2 items (i.e., coping skill and frustration tolerance) of the RTES did not have the same construct as the other items in a previous study [6], we still rephrased them (“Willing to take the therapist's advice to correct his or her movements or other performances” and “Can tolerate discomfort during therapeutic activities”) and added them to the OTES draft-1 because they seemed to reflect patients' engagement in occupational therapy. We further simplified the descriptions of seven items by keeping the core question such that each item contained only one question. Because two of the seven simplified items each contained two valuable questions, we split them into four items. In total, the OTES draft-1 had 17 items.

In step 2 (expert committee review), eight occupational therapists reviewed the OTES draft-1. The experts added three new items (i.e., “Executes at least one home program or bedside activity recommended by the therapist,” “Continues practicing in a wrong way after therapist's instruction,” and “Attends the therapeutic sessions on time without absence for no reason”). The experts suggested the deletion of four items on proposed behaviors that might not be easy to observe (i.e., “Recognizes their accomplishments of occupational therapy” and “Has sufficient self-efficacy for occupational therapy”) or did not fit the daily clinical contexts of occupational therapy (i.e., “Actively request for more challenging activities” and “Can tolerate discomfort during therapeutic activities”). After deletion of the four items, the remaining 16 items were named the OTES draft-2. Fourteen occupational therapists tested the OTES draft-2 and suggested revisions of the wording, format, and timing to record patients' performance. After revisions, the 16 items of the OTES were validated. All items were rated on a 4-point scale. Based on the content that the item measured, there were two sets of descriptors: frequency and attitude. For items measuring the frequency of behaviors (items 1 to 5), the descriptors were from “never” to “always” (0: never, 1: <50% of the time, 2: ≥50% of time, and 3: always). However, some behaviors (items 6 to 10) are unlikely to be observed in clinics (e.g., never listen to the therapist's instructions carefully). We then adjusted the start point of the lowest score to “sometimes” so that the scale would be rated from “sometimes” to “always” (0: sometimes, 1: about 50% of the time, 2: often, and 3: always). Finally, for items measuring patients' attitude (items 11 and 12), the descriptors were from “resists doing so” to “glad to do so” (0: resists doing so, 1: not willing but still does so, 2: willing to do so, and 3: glad to do so). Before scoring patients' engagement, users needed to observe the patients' behaviors for five consecutive days/sessions.

### 4.2. Phase 2: Validation of the OTES

A total of 22 occupational therapists rated the patients' engagement in occupational therapy programs. The majority of the occupational therapists were female (68.2%), and the average age of the therapists was about 40 years. The average number of years of experience as an occupational therapist was about 17 years. A total of 253 patients with stroke were rated by 22 therapists using the OTES. The majority of the patients were male (65.2%). The average age was about 62 years (SD = 13.2). The mean number of months after stroke was 2.1 months (SD = 1.5). The demographic and clinical characteristics of the occupational therapists and patients are shown in Table 1.

### 4.3. Unidimensionality and Reliability

Twelve of the 16 items of the OTES fit the Rasch model's expectations. The 4 nonfitting items were “Attends the therapeutic sessions on time without absence for no reason,” “Executes at least one home program or bedside activity recommended by the therapist,” “Continues practicing in a wrong way after therapist's instruction,” and “Voluntarily discusses with the therapist the latest personal progress or changes in the patient's condition” (infit MNSQ > 1.58, outfit MNSQ > 1.83). Misfit means that these 4 items and the other items assess different dimensions, so they cannot be summed up to represent the level of engagement in OT. We removed one item at a time and reconducted the Rasch analysis until the infit and outfit MNSQ of the remaining 12 items met our preset criteria (ranging from 0.62 to 1.34, Table 2). The PCA of the residual showed that the unexplained variance of the first dimension was 4.0% (<10%).

The person reliability of the 12-item OTES (OTES) was 0.88. One hundred and eighty-eight patients (74.3%) had values of person reliability > 0.90. The patients who had values of reliability < 0.90 had Rasch scores of the OTES ≥ 6.0 or ≤-6.0.

Because the OTES fits the Rasch model's expectations, we transformed the raw sum scores of the OTES into Rasch interval scores.  Table 3 shows the raw sum scores of the OTES, the corresponding Rasch interval scores, and standard errors. Higher scores imply higher engagement in occupational therapy programs. The Rasch scores, a type of standardized score, ranged from -8.0 to 7.3.

### 4.4. Appropriateness of Response Categories

No items exhibited disordering in step difficulty. All differences between adjacent step difficulties were within 1.4-5.0 logits except item 8 (the difference between steps 1 and 2 was 1.29). We retained the response categories of item 8 for two reasons: (1) the difference was close to 1.4, and (2) we wanted to keep all items on a 4-point scale. The step difficulty for each item of the OTES is listed in Table 2. The item step difficulty ranged from -4.49 to 3.94.

### 4.5. Person-Item Mapping

The mean item difficulty for each item of the OTES is listed in Table 2. The range of patients' engagement in occupational therapy programs (-8.0 to 7.3) was larger than the range of item response difficulty (-5.8 to 4.5). In terms of mean item difficulty, the item “Willing to attempt new or unfamiliar therapeutic activities” was the least observed behavior, and the item “Tries his or her best to participate in all therapeutic activities” was the behavior most often observed.

Three significant gaps were noted. The first gap was between step 3 of item 9 (“Cooperates with the therapist and follows the therapist's instructions”; i.e., a Rasch score of 2.6 on engagement or 2.6 logits) and step 2 of item 3 (“Adopts positive or pleasant attitude towards therapeutic activities”; i.e., 0.8 logits); 25.3% (*n* = 64) of the patients were scored within the gap and had person reliability = 0.963–0.966. The second gap was between step 2 of item 9 (i.e., -1.4 logits) and step 1 of item 5 (“Listens to the therapist's instructions carefully”; i.e., -2.2 logits), where 1.6% of the patients (*n* = 4) scored within the gap with person reliability = 0.973–0.974. The third gap was between step 1 of item 2 (“Sustains attention until the end of one therapeutic activity”; i.e., -3.9 logits) and step 1 of item 11 (“Tries his or her best to participate in all therapeutic activities”; i.e., -5.8 logits), and none of the patients scored within the gap.

No patient had the lowest possible score of the OTES (raw sum score = 0), and 20.2% (*n* = 51) of the patients had the highest possible score (raw sum score = 36). Thus, a significant ceiling effect was found.

### 4.6. Predictive Validity

Pearson's *r* between the OTES and the ADL CAT scores was 0.37 (*p* < 0.001).

## 5. Discussion

This is the first study to develop a rating scale to assess patients' engagement levels in occupational therapy. By revising the items of the RTES and adding the recommendations of occupational therapists and experts, we developed a draft of the OTES with 16 items. Rasch analysis was used to determine the final OTES version with 12 items and a 4-point scale.

In validating the data-model fitting, we found that the infit and outfit MNSQ of the final 12 items of the OTES were within the acceptable range (0.6–1.4) [21]. These results indicated that all 12 items of the final OTES fitted the assumptions of the Rasch model and were unidimensional. The four removed items (i.e., being on time, doing home programs, improper practice, and discussion with therapists) were thought to be components of “engagement” based on occupational therapy experience; however, the results showed that these items did not fit the Rasch model's assumptions. The reason might be that these four items may be influenced by caregivers, patients' progress in recovery, or other factors that are separate from the construct of engagement. Thus, we kept only the 12 fitting items.

To validate the unidimensionality of the OTES, in addition to the Rasch model fitting, the PCA of the residuals were calculated and found to be acceptable (no other dimensions explained >10% variance of residuals) [22]. These results demonstrated that the unidimensionality of the OTES was highly supported. Therefore, the score of each item in the OTES can be summed up to represent a person's engagement level. A higher sum score indicates a higher level of engagement. Additionally, the results showed that the OTES contained proper response categories and that the items of the OTES matched most participants' engagement levels. Thus, the OTES appears applicable to the assessment of engagement in patients with stroke who are receiving occupational therapy.

Because the 12 items of the OTES fit the Rasch model's assumptions, we can transform the raw sum scores of the OTES into Rasch scores (an interval scale). In comparison with the raw sum score of the OTES (an ordinal scale), the Rasch score of the OTES has at least two advantages. First, for use in clinical contexts, the Rasch score is useful for quantifying differences and changes in engagement level because the Rasch score has equal intervals of adjacent score points. For example, clinicians can demonstrate exactly the amount of change (or difference) in the engagement level of patients, rather than presenting the change as simply higher or lower. Second, for use in research contexts, Rasch scores are more useful than raw sum scores for arithmetic (e.g., multiplication and division), parametric statistical methods, and statistical inference. For example, researchers can compare the means of Rasch scores of the OTES between two groups of patients in different occupational therapy programs and infer the treatment effectiveness. Thus, the Rasch scores provided in our study are useful to clinicians and researchers for quantifying, analyzing, and interpreting patients' OTES scores.

The results showed that the mean person reliability (0.88) of the OTES was higher than the common criterion (0.7) for group comparison. The person reliability represents the level of the standard error of a respondent's ability (i.e., random measurement errors of the engagement estimation in this study), and higher reliability indicates a lower standard error. Particularly, for individual comparisons, such as comparing individual scores of a person's engagement level, the standard needs to be more stringent because the standard error of an individual score is critical for score interpretation. Our results showed that the person reliability of the OTES was close to the criterion (0.9) for individual comparison. Therefore, occupational therapists can employ the Rasch interval scores to compare the engagement in occupational therapy within an individual patient (e.g., repeated measurements) and between patients with stroke.

We further reviewed the distribution of the person reliability of the patients. The results showed that about 75% of the participants had person reliability > 0.90. Those having person reliability < 0.90 had Rasch scores of the OTES ≥ 6.0 or ≤-6.0. However, a patient with a Rasch score of the OTES ≥ 6.0 would have strong engagement in occupational therapy. For such a patient, clinicians may not need to differentiate the strength of engagement. If a patient's engagement in occupational therapy programs is sufficiently strong, improving the patient's occupational therapy engagement will be of little concern. On the other hand, if a patient has an OTES score≤−6.0, the main issues are to identify what is going wrong and to address the issue. Clinicians or even researchers would not prioritize the determination of the strength of such a patient's engagement. Thus, the 12 items of the OTES appear sufficient for assessing the patients' level of engagement in occupational therapy for research and clinical purposes.

In terms of the person-item mapping in this research, the range of item response difficulties was smaller than that of the participants' engagement levels. Additionally, ceiling effects were noted, indicating that the items of the OTES for assessing rather high engagement were insufficient for the participants. The ceiling effects may have resulted from two factors: a selection bias and the intention of the patients. The selection bias may have occurred because patients with low engagement may have refused to participate in this study. The intention of almost all patients is recovery, so they tend to engage in occupational therapy programs. Fortunately, differentiating the various levels of patients with high engagement is not the main issue in clinical settings. Patients with suboptimal engagement levels are likely to have sufficient engagement to facilitate their recovery.

Three substantial gaps existed. A substantial gap means that the distance between two adjacent item response categories is so large that the levels of patients within the range may not be estimated reliably. In other words, in a gap, the Rasch reliability decreases and a patient's level of engagement cannot be well distinguished. The first gap (0.8 to 2.6 logits) was of concern because 25.3% of the participants' estimated engagement levels fell within this gap. The second (-2.2 to -1.4 logits) and third (-5.8 to -3.9 logits) gaps may not be of concern because few participants (1.6%) had scores within these two gaps. However, the average person reliabilities of patients in the first and second gaps were about 0.96 and 0.97, respectively. The very high Rasch reliability of the participants should ease concerns about the gaps. Many items located on both sides of the first and second gaps may contribute to the high person reliabilities of the patients in these two gaps. Thus, the person-item mapping further supports the result that the items of the OTES are sufficient for assessing the levels of engagement of patients with stroke.

We found that the OTES scores had substantial association with those of the ADL CAT assessed at 2 months after discharge (Pearson's *r* = 0.37). This finding indicates that the predictive validity of the OTES is acceptable. In a previous study, several well-examined factors (e.g., motor function and cognition) may influence patients' ADL performance; patients' engagement in occupational therapy seems to be an important but rarely examined factor that affects their ADL performance [12, 26]. The relationship between engagement and ADLs may not be causal in nature. Although we expect that high levels of engagement will contribute to improvement in functioning, it is also possible that this relationship reflects a greater capacity to become engaged among individuals who are highly functioning. We still refer to this as predictive validity because the engagement ratings are associated with ADLs measured at a future time point. We recommend that therapists use the OTES to assess patients' engagement in occupational therapy in their daily practice. Further studies are needed to explore possible factors affecting engagement in occupational therapy. The results would contribute to the management of patients' engagement in occupational therapy and might further improve the management of patients' ADL function.

In a clinical practice, the score of each item and the sum score of the OTES can be applied for different purposes. Each item of the OTES can be taken as a specific criterion to observe stroke patients' engagement in occupational therapy programs. Through the score of each item, therapists would be able to monitor the criteria that patients fulfill, rather than recording the patient's engagement on a rough single scale, such as low, medium, and high. The sum score of the OTES can reflect patients' preferences for different treatments or styles of intervention. If a patient has low scores of the OTES, therapists can discuss the reasons with the patients to modify the therapeutic programs and thereby provide more client-centered programs. Moreover, the sum scores of the OTES could be used as a predictor of patients' outcomes. Patients with low scores of the OTES may have low motivation for practicing activities that their therapists provide, which might result in poor outcomes [1, 2]. Thus, a low score of the OTES can alert occupational therapists to figure out the causes of decreased patient engagement and try to deal with them in a timely manner. However, the cutoff points of low engagement and acceptable engagement need to be verified in future studies.

### 5.1. Limitations and Directions for Future Research

Our study has three limitations. The first is that we recruited only hospitalized patients with stroke onset within 6 months. Such a recruitment bias may hamper the generalization of the results to all patients with stroke receiving occupational therapy. Future research recruiting inpatients and outpatients with various intervals after stroke onset to verify our results is warranted. The second limitation is that we did not recruit patients with severe cognitive and/or communication deficits because we were unsure whether they would be unable or unwilling to follow therapists' instructions. The third limitation is that we used Pearson's correlation coefficient to estimate the predictive validity of the OTES, which might have over- or underestimated the relationship between engagement and patients' ADL performance. The predictive power of the OTES would be better examined using regression analysis to control for confounders (e.g., motor and cognitive impairments). Unfortunately, we could not collect sufficient data in the medical records related to other predictors of patients' ADL performance, such as motor impairment severity, presence of depression, and cognitive impairment at admission. Therefore, we could not conduct regression analysis. Future studies could use different statistical methods to validate our results.

## 6. Conclusion

The OTES was developed through reviewing similar scales, considering experts' opinions, and field testing. The OTES is unidimensional and has sufficient person reliability and predictive validity in patients with stroke. The OTES could help clinicians and researchers to determine accurately the levels of engagement of patients with stroke. Future researchers can identify the factors influencing the scores of the OTES to improve the integrity of the theories of engagement and motivation.

## Figures and Tables

**Table 1:** Demographic and clinical characteristics of the patients and occupational therapists rating the OTES.

Characteristic	
*Occupational therapists rating the OTES* (*N* = 22)	
Gender (male/female)	7/15
Age (years); mean ± SD	40.2 ± 7.2
Degree of education (bachelor/master)	15/7
Years working as an occupational therapist; mean ± SD	17.4 ± 7.5
*Patients* (*N* = 253)	
Gender (male/female)	165/88
Age (year)	62.3 ± 13.2
Level of education	
Not educated	20 (7.9%)
≤6 years	62 (24.5%)
≤9 years	96 (38.0%)
10–12 years	27 (10.7%)
13–16 years	33 (13.0%)
>16 years	11 (4.3%)
Missing	4 (1.6%)
Months after stroke; mean ± SD	2.1 ± 1.5
Side of brain lesion	114/132/7
Left	114 (45.1%)
Right	132 (52.1%)
Both	7 (3.8%)
Incidents of stroke (1/≥2)	205/48
Aphasia: comprehension impairment	
Normal	172 (68.0%)
Mild	54 (21.3%)
Moderate	26 (10.3%)
Severe	1 (0.4%)
Aphasia: expression impairment	
Normal	147 (58.1%)
Mild	51 (20.2%)
Moderate	42 (16.6%)
Severe	13 (5.1%)
Raw OTES total scores; mean ± SD	39.3 ± 8.0

**Table 2:** The infit and outfit mean square (MNSQ) statistics, mean item difficulties, and standard error (SE) of mean difficulty and step parameters of the occupational therapy engagement scale (OTES).

Item	Infit MNSQ	Outfit MNSQ	Mean difficulty	SE	Step 1	Step 2	Step 3
(1) Commits in therapy activities without being urged	1.01	0.96	0.06	0.14	-2.72	-0.30	3.02
(2) Sustains attention until the end of one therapeutic activity	0.99	0.98	-0.16	0.14	-3.78	0.13	3.65
(3) Adopts positive or pleasant attitude towards therapeutic activities	1.29	1.33	0.49	0.14	-3.95	0.29	3.66
(4) Is easily encouraged by the therapist to engage more in therapeutic activities	0.82	0.72	-0.12	0.14	-3.01	0.29	2.72
(5) Tries his or her best to participate in all therapeutic activities	0.97	0.96	-1.34	0.15	-4.49	0.55	3.94
(6) Listens to the therapist's instructions carefully	0.86	0.87	0.42	0.14	-2.58	-0.77	3.34
(7) Correctly executes the therapeutic activities designed by the therapist without arbitrary adjustment of the activity content	1.01	1.05	-0.29	0.15	-3.08	-0.71	3.79
(8) Is willing to take the therapist's advice to correct his or her movements or other performances	0.83	0.78	0.06	0.14	-2.32	-1.03	3.35
(9) Cooperates with the therapist and follows the therapist's instructions	0.76	0.62	-0.59	0.15	-2.33	-0.85	3.18
(10) Completes the number of times or duration of activity recommended by the therapist before the end of each therapy session	1.34	1.20	-0.19	0.14	-2.11	-0.64	2.75
(11) Accepts physically or mentally challenging activities	0.89	0.87	0.77	0.14	-3.42	-0.28	3.70
(12) Is willing to attempt new or unfamiliar therapeutic activities	1.11	1.10	0.87	0.14	-3.20	-0.45	3.65

^†^The items “Attends the therapeutic sessions on time without absence for no reason,” “Executes at least one home program or bedside activity recommended by the therapist,” “Continues practicing in a wrong way after therapist's instruction,” and “Voluntarily discusses with the therapist the latest personal progress or changes in the patient's condition” were not included because their infit and outfit MNSQ were beyond the preset criteria (infit MNSQ > 1.58, outfit MNSQ > 1.83). ^‡^Descriptors for each item—items 1 to 5: 0 (never), 1 (<50% of the time), 2 (≥50% of time), and 3 (always); items 6 to 10: 0 (sometimes), 1 (about 50% of the time), 2 (often), and 3 (always); and items 11 and 12: 0 (resists doing so), 1 (not willing but still does so), 2 (willing to do so), and 3 (glad to do so).

**Table 3:** Raw sum scores, Rasch scores, and standard errors of the occupational therapy engagement scale (OTES).

Raw sum score	Rasch score	Standard error
0	-8.0	1.7
1	-6.0	1.2
2	-4.9	0.8
3	-4.3	0.7
4	-3.9	0.6
5	-3.5	0.6
6	-3.2	0.6
7	-2.9	0.5
8	-2.6	0.5
9	-2.4	0.5
10	-2.1	0.5
11	-1.9	0.5
12	-1.7	0.5
13	-1.4	0.5
14	-1.2	0.5
15	-1.0	0.5
16	-0.7	0.5
17	-0.5	0.5
18	-0.2	0.5
19	0.1	0.5
20	0.3	0.5
21	0.6	0.5
22	0.9	0.6
23	1.2	0.6
24	1.6	0.6
25	1.9	0.6
26	2.2	0.6
27	2.5	0.6
28	2.8	0.6
29	3.2	0.6
30	3.5	0.6
31	3.8	0.6
32	4.2	0.6
33	4.6	0.7
34	5.2	0.8
35	6.0	1.1
36	7.3	1.9

^†^No patients had scores of zero or within the range of 2–5; thus, we applied the maximum likelihood method to simulate the Rasch scores of the corresponding raw sum scores.

## Data Availability

The data used and/or analyzed during the current study are available from the corresponding author on reasonable request.
